# Supplementary material for: Vitamin D Modified DSS-Induced Colitis in Mice via STING Signaling Pathway
Source: Biology (Basel). 2025 Jun 18;14(6):715. doi: 10.3390/biology14060715 (PMC12190092; doi:10.3390/biology14060715)
Supplement: Supplementary file 1 [file biology-14-00715-s001.zip › Supplementary Materials S1: 16s-report of gut microbiota/Groups/betadiv/pcoa_bray_analysis/Pcoa_bray23_boxplot.pdf]

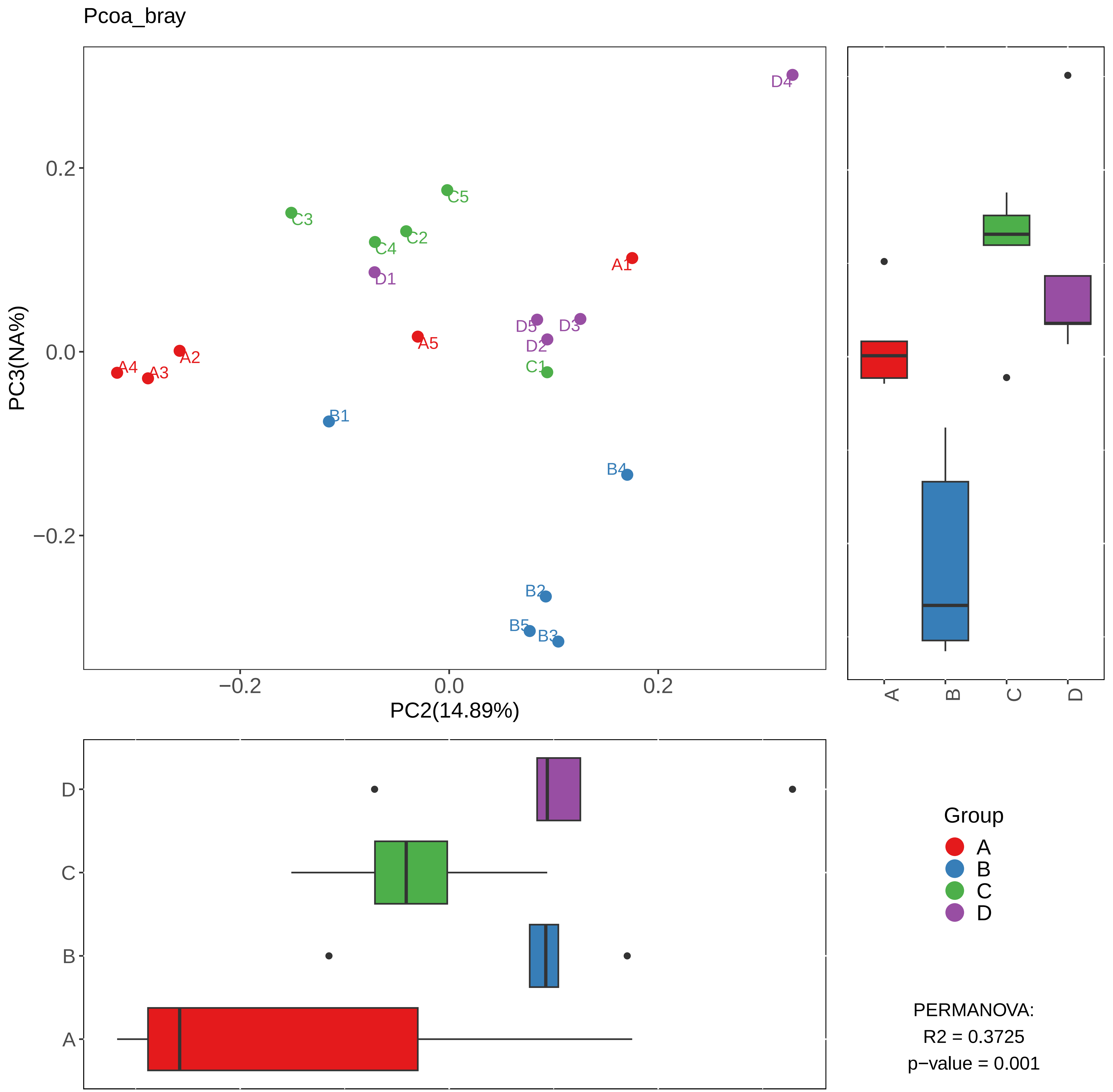

Pcoa_bray
PC3(NA%)
PC2(14.89%)
0.2
0.0
−0.2
−0.2
0.0
0.2
A1
A2
A3
A4
A5
B1
B2
B3
B4
B5
C1
C2
C3
C4
C5
D1
D2
D3
D4
D5
A
B
C
D
D
C
B
A
Group
A
B
C
D
PERMANOVA:
R2 = 0.3725
p−value = 0.001
